# The latent profile of health literacy among older adults patients with chronic disease comorbidity and its association with self-management behaviors

**DOI:** 10.3389/fpubh.2026.1880321

**Published:** 2026-09-11

**Authors:** Jianrui Guo, Bo Ma, Wenjing Xue

**Affiliations:** 1 Jinzhou Medical University, Jinzhou, China; 2 Liaoning Provincial People's Hospital, Shenyang, China

**Keywords:** chronic disease, health literacy, latent profile analysis, older adults, self-management behaviors

## Abstract

**Background:**

Chronic diseases pose a heavy worldwide public health burden. China is both one of the fastest-aging nations and the one with the largest older adults population. Among its older adults, chronic disease comorbidity is highly common and seriously harms both health outcomes and quality of life. Successful disease management requires not just clinical care but also adequate health literacy in everyday settings. Nevertheless, health literacy levels in Chinese older adults continue to be relatively low. Clarifying the different health literacy subgroups among multimorbid older adults individuals and how they relate to self-management behaviors is critical for designing targeted programs and supporting healthy aging.

**Objective:**

This study sought to uncover latent health literacy classes in older adults with multimorbidity, characterize their features and determinants, and evaluate their link to self-management behaviors.

**Methods:**

A convenience sampling method was used to enroll 302 older adults with multiple chronic conditions at Liaoning Provincial People’s Hospital in China from November 2025 to January 2026. The collected data covered sociodemographic variables, Health Literacy Measurement Scale for Chronic Disease Patients scores, and Chronic Disease Self-Management Behavior Scale scores. Latent profile analysis (LPA) was performed with Mplus 8.3, and univariate analyses together with multivariate logistic regression were applied to determine factors linked to health literacy profiles.

**Results:**

The average health literacy score was 84.37 ± 15.59. Three distinct classes emerged: “low literacy–limited information comprehension” (18.5%, *n* = 56), “moderate literacy-physician-dependent communication” (42.6%, *n* = 129), and “high literacy-autonomous and efficient decision-making” (38.9%, *n* = 117). According to multivariate logistic regression, sex, age, residence location, education level, Alcohol consumption, and illness duration significantly associated with class belonging.

**Conclusion:**

Overall, health literacy levels in older multimorbid adults are relatively low in this sample, especially regarding communication and interactive skills. Considerable heterogeneity exists across subgroups, and self-management behaviors differ markedly among the identified profiles. Profile-specific, tiered intervention approaches are necessary to boost health literacy and enhance self-management in this population.

## 1 Introduction

Chronic diseases, defined by their prolonged duration and typically gradual progression, represent a significant threat to global public health. The World Health Organization (WHO) reports that these conditions are responsible for approximately 41 million deaths annually, which constitutes a considerable 71% of total global mortality (1). Chronic noncommunicable diseases, such as cardiovascular and cerebrovascular diseases, malignant neoplasms, and diabetes mellitus, account for 88% of fatalities in China, significantly endangering public health (2). The WHO characterizes “comorbidity” as the coexistence of two or more chronic health issues requiring continuous medical attention (3). People with comorbidity have notably elevated mortality and disability risks, diminished health-related quality of life, and increased healthcare usage and caregiving responsibilities compared to individuals with only one condition (4, 5). This burden is particularly pronounced among older adults.

Projections suggest that by 2030, there will be 1.4 billion people aged 60 or older worldwide (6). China exemplifies this global trend more than most countries, given that it has the world’s largest population and is experiencing particularly fast demographic aging. The most recent national figures show that Chinese citizens 60 years of age or above make up 18.7% of the country’s total population (7); by roughly 2035, that figure is projected to surpass 400 million, indicating that China will soon enter a phase of deep population aging (8). Available epidemiological data indicate that 83.1% of older Chinese adults suffer from at least one chronic illness (9), and having multiple such conditions is extremely common. Chronic diseases often appear together and influence one another, resulting in diverse and intricate multimorbidity profiles (10). In fact, more than a third of China’s older adults population is managing two or more chronic diseases at once (11). Collectively, these trends place considerable strain on the nation’s healthcare system in terms of chronic disease prevention, management, and impact reduction. This situation strongly highlights the need to examine changeable factors—health literacy and self-management behaviors, for example—that could help address the problem.

Older adults in China face a high burden of comorbidity. Because chronic illnesses are long-lasting and tend to worsen over time, managing them effectively requires more than occasional hospital visits—it largely depends on how consistently patients carry out daily health-related activities (12). Self-management refers to the various actions people take to control their illness, avoid or lessen complications, and reduce negative impacts on their social life and mental health (13). A person’s health literacy serves as a key foundation for successful, long-term self-management. Ren et al. conceptualized health literacy as the ability to find, understand, and use basic health information and services to make sound health decisions (14). Older adults with multiple chronic conditions often face complicated medication regimens, including the use of many drugs at once. For them, health literacy plays an important connecting role between what they know about health and what they actually do. This connection directly affects how well they manage their conditions, their clinical outcomes, and their overall quality of life. Existing research backs up this link. Lanxin W. and colleagues (15) found that older adults with multimorbidity who have stronger health literacy show better disease awareness and more adaptive coping skills, which in turn boosts their ability to manage their own conditions. Likewise, Ren et al. (16) carried out a multicenter study and concluded that health literacy strongly and positively predicts how well this population can handle self-management tasks. Previous work also consistently shows that people who manage their chronic conditions well tend to have a higher quality of life, and health literacy appears to be an important changeable factor driving this relationship (14).

Although health literacy is crucial, the overall level among the population remains suboptimal. The situation is particularly concerning among older adults, many of whom face difficulties in communicating with physicians, adhering to medication regimens, managing chronic conditions, and adopting health-promoting behaviors—consistent with previous research findings (2). In short, health literacy is a key factor in successful disease management, directly enhancing self-management capabilities and improving quality of life for patients with chronic illnesses.

However, health literacy is not uniformly distributed among older adults but is profoundly influenced by multiple factors. Studies show that advancing age consistently acts as an independent risk factor for lower health literacy—age-related cognitive decline significantly limits older individuals’ ability to access and use health information (17). Regarding gender, findings remain mixed: some studies suggest women have higher health literacy due to greater involvement in caregiving roles and more frequent use of healthcare services, while other evidence indicates that gender effects vary across age groups (18). Educational attainment is one of the strongest predictors, with higher levels of education associated with better health literacy (19). Additionally, the duration of chronic illness can influence health literacy through accumulated healthcare experiences—the longer the disease course, the more opportunities patients have to gain experiential knowledge through ongoing disease management (20). Notably, most of these studies employ traditional variable-centered analytical approaches, which are inadequate for uncovering the underlying heterogeneous structure of health literacy in older adults with multiple comorbidities. Therefore, it is necessary to adopt person-centered latent profile analysis to explore potential subtypes of health literacy and their characteristic differences within this population (21). Guided by this framework, the present study applies latent profile analysis to: (a) identify distinct profiles of health literacy among older adults with multiple chronic conditions; (b) examine how different health literacy profiles are associated with self-management capacity; and (c) provide actionable evidence for developing targeted intervention strategies.

## 2 Materials and methods

### 2.1 Study population

Participants were recruited through convenience sampling from a tertiary hospital in Shenyang between November 2025 and January 2026. To be eligible for the study, individuals had to meet all of the following criteria: (1) being 60 years of age or older; (2) having received a diagnosis of at least two chronic diseases (including hypertension, diabetes, dyslipidemia, heart disease, stroke, and 15 other chronic conditions); (3) being fully conscious and able to read or communicate at a basic level; (4) having had their chronic condition(s) for 6 months or longer; and (5) providing informed consent and agreeing to take part voluntarily. People were excluded if they had: (1) severe visual, hearing, or mental impairments; (2) acute or critical illnesses accompanied by major complications or organ failure; or (3) already enrolled in other similar studies.

The core analytical method of this study is latent profile analysis (LPA), whose power requirements differ fundamentally from those of regression-based approaches. Following Tein, Coxe, and Cham (22), statistical power in LPA is primarily determined by class separation (Cohen‘s d) and the number of indicators. Their simulations demonstrated that when key indicators exhibit an effect size of 0.8 or above and the number of indicators is sufficient (≥10), a sample size of 250 yields power exceeding 0.80 for the BIC and BLRT to identify the correct number of classes. In the present study, we included 24 indicators, which exceeds the recommended threshold. A total of 305 questionnaires were distributed, and 302 were returned with valid responses, yielding a valid response rate of 99.02%. With a total sample of 302, our study meets the statistical requirements for detecting the correct number of latent profiles. Nylund-Gibson and Choi (23) further reviewed LPA simulation studies and suggested that sample sizes between 300 and 1,000 are generally adequate, which further supports the adequacy of our sample size. For descriptive purposes, Cohen’s d values for the core indicators between adjacent classes are presented in Supplementary Table 1, with the majority exceeding 0.80.

The study was approved by the Medical Ethics Committee of Jinzhou Medical University (approval No. JZMULL2025480). Every participant gave voluntary consent before taking part.

### 2.2 Survey instruments

#### 2.2.1 General information questionnaire

Researchers developed a general information questionnaire informed by a literature review and team discussions. The questionnaire included items on sex, age, body mass index (BMI), marital status, current residence, employment status, educational attainment, per capita monthly household income, payment method, number of children, smoking status, drinking status and duration of chronic diseases.

#### 2.2.2 Health Literacy Measurement Scale for Chronic Disease Patients

Health literacy was assessed using the Health Literacy Measurement Scale for Patients with Chronic Diseases, developed by Jordan et al. (24) and translated by Sun and Haolin (25). The scale consists of 24 items categorized into four dimensions: information acquisition ability, communication and interaction ability, willingness to enhance health, and willingness to seek financial support. Each item was rated on a 5-point Likert scale, with scores ranging from 1 to 5, leading to total scores between 24 and 120. Higher scores indicated superior health literacy levels. The scale demonstrated a Cronbach’s α coefficient of 0.918, while in this research, Cronbach’s α was 0.935, with a KMO value of 0.918 and a sphericity test (*p* < 0.001). The Cronbach’s α coefficients for the four subscales—information acquisition ability, communication and interaction ability, willingness to enhance health, and willingness to seek financial support—were 0.917, 0.784, 0.904, and 0.964, respectively (Supplementary Tables 3, 4).

#### 2.2.3 Chronic Disease Self-Management Behavior Scale

The Chronic Disease Self-Management Behavior Scale, adapted by Chinese scholar Qiao Han (26), comprises five dimensions: diet, exercise, emotional management, medication adherence, and social support, with 20 items. It employs a 5-point Likert scale, yielding total scores ranging from 20 to 100. Scores of 80–100 indicate high self-management levels, 60–79 indicate moderate self-management, and 20–59 indicate poor self-management, with lower scores reflecting poorer self-management. The scale’s Cronbach’s α coefficient was 0.957. In this study, Cronbach’s α was 0.783, the subscale reliabilities ranged from 0.520 to 0.725 across the five dimensions. The KMO value was 0.775, and the sphericity test yielded *p* < 0.001.

### 2.3 Data collection methods

We gathered data through both online and paper-based surveys. Before starting, two trained staff members gave patients a standardized explanation of what the study was about. Once we had obtained each participant’s informed consent, we went over the questionnaire’s content and how to complete it in detail. If a patient had physical difficulty writing or using a digital device, a family member could serve as a scribe. In such cases, the patient remained present throughout the entire process, and the family member recorded the patient‘s own answers without paraphrasing or adding any personal interpretation. The responses were dictated by the patient themselves, ensuring that the content reflected the patient’s own perspectives and understanding.

Completing the survey took about 8 to 15 min. Participation was entirely voluntary and anonymous. After participants finished, paper questionnaires were collected in person by the research staff, who reviewed each one individually so that any missing information could be added right away. Electronic questionnaires were tracked live through the research platform. Whenever responses were unclear, we contacted the participants again to double-check their answers. For paper-based questionnaires, all data were entered twice and cross-checked to ensure quality and completeness.

### 2.4 Statistical methods

All statistical analyses were carried out using Mplus 8.3 and SPSS 27.0. For latent profile analysis (LPA), we entered the individual item scores from the health literacy scale as manifest variables in Mplus. We fitted models specifying one to four latent classes to classify the multimorbid older adult patients and then selected the model that provided the best fit. Model fit was evaluated using the Akaike information criterion (AIC), Bayesian information criterion (BIC), sample size-adjusted BIC (aBIC), and entropy. The Lo–Mendell–Rubin adjusted likelihood ratio test (LMR) and the bootstrap likelihood ratio test (BLRT) were used to compare models. Lower values of AIC, BIC, and aBIC indicate a better fit. Entropy is a measure of classification accuracy that ranges from 0 to 1, with scores closer to 1 meaning more precise classification. An entropy value of 0.8 or higher corresponds to classification accuracy greater than 90%. The LMR and BLRT compare the fit of competing models; a statistically significant *p*-value means the k-class model fits better than the k-1-class model. Average posterior probabilities above 0.70 are considered acceptable for class assignment, while values above 0.85 reflect high-quality classification (11, 12).

Latent profile analysis (LPA) was conducted using Mplus version 8.3. The model was estimated using robust maximum likelihood (MLR) estimator, which provides standard errors and fit statistics that are robust to non-normality. To avoid local maxima and ensure convergence to the global log-likelihood solution, we specified 5,000 random starts in the initial stage and retained the 1,000 iterations with the best log-likelihood values for final optimization. Convergence of each model was verified by (a) replication of the best log-likelihood value across multiple random starts, and (b) stability of parameter estimates across final stage iterations. All models assumed equal variances across latent classes (i.e., homoscedasticity) and zero covariances among the 24 indicator items (i.e., conditional local independence), consistent with the default LPA specification in Mplus. To evaluate the tenability of the local independence assumption, we inspected the standardized residual correlations from the fitted 3-class model. Residual dependencies were considered acceptable if they were mainly confined to item clusters within the same theoretical dimensions, as this pattern reflects expected content overlap rather than a substantive violation of the assumption.

SPSS 27.0 was utilized for statistical analysis. Quantitative data with normal distribution were shown as mean ± standard deviation, while non-normally distributed data were displayed as median and interquartile range. Categorical variables were analyzed using the chi-square test or Fisher’s exact test, as appropriate. Since the number of potential levels for health literacy categories in older adult patients with chronic disease comorbidity exceeded two, unordered multinomial logistic regression analysis was employed to examine factors associated with health literacy in this population, with a significance level of α = 0.05. For the comparison of self-management behavior scores across the three health literacy profiles (Table 1), analysis of covariance (ANCOVA) was employed, controlling for age, sex, education, residence, and disease duration as covariates. Post-hoc pairwise comparisons were performed using the Bonferroni correction to adjust for multiple comparisons. Data are presented as adjusted mean ± standard error.

**Table 1:** Model fit indices for latent profile analysis of health literacy among older adults patients with chronic disease comorbidities (*n* = 302).

Model	AIC	BIC	aBIC	Entropy	*p*		Class probability (%)
					LMR	BLRT	
Model 1	20347.051	20525.152	20372.922				
Model 2	18158.533	18429.395	18197.878	0.950	<0.001	<0.001	49.81/50.19
Model 3	17603.227	17966.849	17656.047	0.941	0.0079	<0.001	18.47/42.61/38.92
Model 4	17119.972	17576.354	17186.265	0.948	0.2185	<0.001	21.14/21.93/25.85/31.09

AIC, Akaike Information Criterion; BIC, Bayesian Information Criterion; aBIC, sample size-adjusted BIC; Entropy, information entropy; LMR, Lo–Mendell–Rubin adjusted likelihood ratio test; BLRT, Bootstrap likelihood ratio test.

## 3 Results

### 3.1 Common method bias test

To check for common method bias, we applied Harman’s single-factor test. We ran an unrotated exploratory factor analysis on the 44 items taken from the existing scales. This analysis extracted 11 factors with eigenvalues above 1, which together accounted for 67.78% of the total variance. The first factor explained 29.09% of the variance, which did not reach the commonly used cutoff of 40%. Based on this result, we concluded that common method bias was not a serious concern in this study.

### 3.2 General characteristics of older adults patients with chronic co-morbidities

Out of 305 distributed questionnaires, 302 were completed validly, giving a response rate of 99.02%. The largest age group was 60–70 years, accounting for 56.6% of the sample. Among the 302 participants, 60.9% (*n* = 184) were male, 83.8% (*n* = 253) were married, and 77.5% (*n* = 234) lived in urban areas. Regarding education, 48.3% (*n* = 146) had completed junior high school or technical secondary school. Monthly household income fell between 1,001 and 3,000 RMB for 62.3% (*n* = 188) of the participants. Retired workers made up 69.9% (*n* = 211) of the sample. In addition, 64.2% (*n* = 194) had only one child. Regarding lifestyle factors, 70.5% (*n* = 213) were non-smokers and 66.6% (*n* = 202) were non-drinkers. Employee medical insurance was held by 54.6% (*n* = 165) of the participants. Chronic disease duration exceeded 10 years for 45.4% (*n* = 137), and 45.7% (*n* = 138) had a BMI below 24. The remaining general characteristics are shown in Supplementary Table 2.

### 3.3 Categories of health literacy among older adults patients with chronic disease comorbidities

This study conducted a latent profile analysis using the 24 items of the Health Literacy Measurement Scale (HLMS). Starting from a one-class model, the number of classes was progressively increased, and latent profile models for one to four classes were successively fitted. As shown in Table 2, the AIC, BIC, and aBIC values decreased as the number of classes increased, with the decline slowing when transitioning from three to four classes. All models had entropy values exceeding the recommended threshold of 0.8, indicating good classification accuracy.

**Table 2:** Univariate analysis of latent profiles of health literacy among older adults patients with chronic disease comorbidities.

Variable	Category	C1	C2	C3	Test statistic	*p*
					(χ2)	
Age	60–70	18(32.1)	69(53.5)	84(71.8)	25.145	<0.001
	≥71	38(67.9 h	60(46.5)	33(28.2)		
Sex	Male	24(42.9)	82(63.6)	78(66.7)	9.677	0.008
	Female	32(57.1)	47(36.4)	39(33.3)		
Marital status	Married	44(78.6)	102(79.1)	107(91.5)	8.291	0.016
	Divorced/Widowed	12(21.4	27(20.9)	10(8.5)		
Place of residence	Urban	34(60.7)	96(74.4)	104(88.9)	18.444	<0.001
	Rural	22(39.3)	33(25.6)	13(11.1)		
Educational attainment	Primary school or below	32(57.1)	33(25.6)	5(4.3)	86.848	<0.001
	Junior high or technical secondary school	20(35.7)	74(57.4)	52(44.4)		
	High school or above	4(7.1)	22(17.1)	60(51.3)		
Monthly household income per capita	≤1,000	25(44.6)	15(11.6)	8(6.8)	68.177	<0.001
	1,001–3,000	26(46.4)	98(76.0)	64(54.7)		
	≥3,001	5(8.9)	16(12.4)	45(38.5)		
Occupational status	Retired	32(57.1)	86(66.7)	93(79.5)	10.940	0.027
	Non-retired	17(30.4)24(42.9)	43(33.3)	24(20.5)		
Number of children	One	26(46.4)	84(65.1)	84(71.8)	4.02	0.134
	Two or more	30(53.6)	45(34.9)	33(28.2)		
Smoking status	Smoker	16(28.6)	38(29.5)	35(29.9)	10.683	0.005
	Non-smoker	40(71.4)	91(70.5)	82(70.1)		
Alcohol consumption	Drinker	28(50.0)	43(33.3)	30(25.6)	10.097	0.006
	Non-drinker	28(50.0)	86(66.7)	87(74.4)		
Duration of chronic disease	Less than 3 years	14(25.0)	29(22.5)	5(4.3)	27.344	<0.001
	3 to 10 years	26(46.4)	49(38.0)	42(35.9)		
	More than 10 years	16(28.6)	51(39.5)	70(59.8)		
Payment method	Employee medical insurance	37(66.1)	73(56.6)	55(47.0)	5.900	0.052
	Others	19(33.9)	56(43.4)	62(53.0)		
BMI	<24	30(53.6)	59(45.7)	49(41.9)	4.641	0.326
	24–28	23(41.1)	52(40.3)	49(41.9)		
	>28	3(5.4)	18(14.0)	19(16.2)		

C1: Low literacy-Limited information comprehension type; C2: Moderate literacy-Physician-dependent communication type; C3: High literacy-Autonomous and efficient decision-making type.

The LMR test and BLRT were applied to compare models with k versus k-1 classes. In the transition from the three-class to the four-class model, the LMR test was non-significant (*p* = 0.2185), indicating that the more complex four-class solution did not provide a statistically significant improvement in fit over the three-class model. Although the BLRT was significant (*p* < 0.001) for this comparison, given the known over-sensitivity of the BLRT to trivial improvements in moderately large samples, methodological guidelines recommend prioritizing the LMR test result when the two tests are in conflict (27). Notably, both the LMR and BLRT tests were significant for the two-class versus one-class and three-class versus two-class comparisons (*p* < 0.05). Thus, the non-significant LMR test served as the statistical justification for retaining the three-class solution. This statistical decision was further corroborated by the diminishing decline in AIC, BIC, and aBIC values (i.e., an elbow point) and the clinical interpretability of the three class profiles. Accordingly, the three-class model was selected as the optimal solution for older adult patients with chronic disease multimorbidity.

The three-class model demonstrated excellent classification quality, with an entropy value of 0.941. The average posterior probabilities for the three classes were 0.964, 0.969, and 0.986, respectively, all exceeding the recommended threshold of 0.85 for good classification quality. Examination of the standardized residual correlations revealed that the vast majority of residual correlations were below |0.10|, supporting the local independence assumption. Only a few item pairs within the same theoretical dimensions showed slightly elevated residuals, which likely reflects content overlap rather than a substantive violation of the assumption. The overall residual pattern did not materially distort the class solution, as further supported by the high entropy value (0.941).

**Figure 1:**
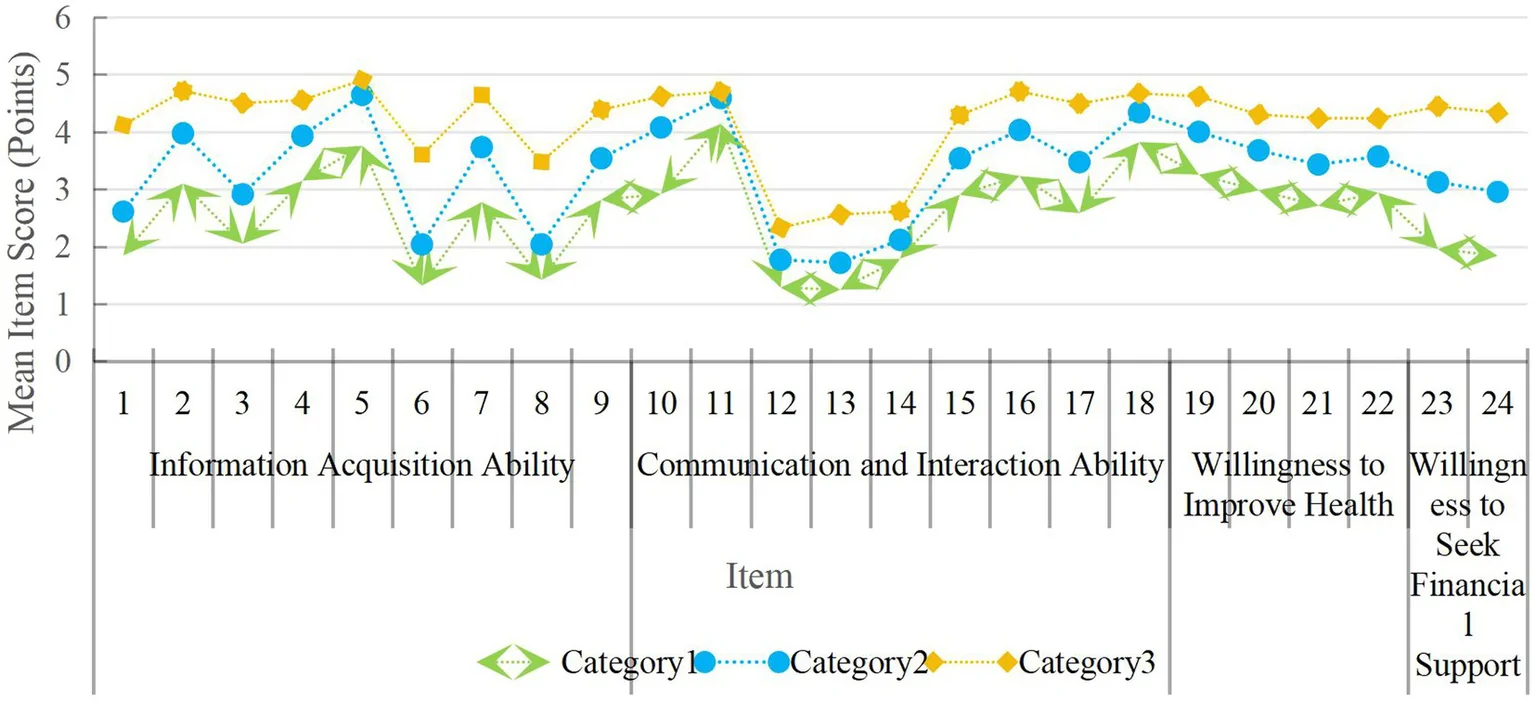
Latent profiles of health literacy among older adults patients with chronic disease comorbidity.

Using the latent profile plot (Figure 1), we examined the patterns of each class and gave them names based on their unique features. Class 1 (C1) contained 56 patients (18.5%) and had a mean health literacy score of 61.80 ± 7.31. As seen in Figure 1, this class scored the lowest on all 24 items, with especially low scores on items 1, 6, 8, 12, 13, and 24. We therefore labeled this class the “low literacy–limited information comprehension” type. Class 2 (C2) included 129 patients (42.6%) and had a mean health literacy score of 79.92 ± 6.05. Items 2, 5, 11, and 18 received higher scores in this group, reflecting strong compliance with medical advice and a tendency to follow others’ guidance. Accordingly, we named this class the “moderate literacy-physician-dependent communication” type. Class 3 (C3) consisted of 117 patients (38.9%) and had the highest mean health literacy score among the three groups (100.08 ± 6.95). This class scored highest on all items, and its scores on items 3, 6, 8, 13, and 17 were notably higher than those of the other two classes. Consequently, we termed this the “high literacy–autonomous and efficient decision-making” type.

### 3.4 Univariate analysis of latent categories of health literacy in older adults patients with chronic disease comorbidities

As shown in Table 3, the three health literacy classes differed significantly across several sociodemographic and disease-related variables. Specifically, we observed significant associations (*p* < 0.05) with age, sex, marital status, place of residence, Educational attainment, average monthly household income, occupation, Smoking status, Alcohol consumption, and Duration of chronic disease.

**Table 3:** Variable assignment.

Variable	Assignment
Sex	Female as reference, Male = 1
Age	60–70 years as reference, ≥71 years (0,1)
Marital status	Divorced/Widowed as reference, Married (1,0)
Place of residence	Urban as reference, Rural (0,1)
Educational attainment	High school or above as reference, Primary school or below (1,0,0); Junior high or technical secondary school (0,1,0)
Monthly household income per capita (¥)	≥3,001 as reference; ≤1,000 (1,0,0); 1,001–3,000 (0,1,0)
Occupational status	Non-retired as reference, Retired (1,0)
Smoking status Alcohol consumption	Non-smoker as reference, Smoker (1,0) Non-drinker as reference, Drinker (1,0)
Duration of chronic disease	3–10 years as reference, <3 years (1,0,0), >10 years (0,0,1)

### 3.5 Multivariate analysis of latent categories of health literacy among older adults patients with chronic disease comorbidities

We carried out multivariate logistic regression using the “low literacy–limited information comprehension” class as the reference category. The other two literacy classes served as the outcome variables. Predictors included all variables that had been statistically significant in the univariate analyses. Dummy coding was applied to categorical variables; the coding scheme is presented in Table 3. The results of the multivariate analysis are presented in Table 4.

**Table 4:** Multivariate logistic regression analysis of health literacy profiles among older adults patients with chronic disease comorbidities (*n* = 302).

Group	Variable	β	SE	Waldc^2^	*p*	OR	95%CI	
2	Sex (ref: Female)							
	Male	1.441	0.474	9.253	0.002	4.227	1.67	10.7
	Age(years) (ref: ≥71 years)							
	60–70	0.861	0.42	4.191	0.041	2.364	1.037	5.389
	Marital status (ref: Divorced/Widowed)							
	Married	−0.808	0.507	2.534	0.111	0.446	0.165	1.205
	Place of residence (ref: Rural)							
	Urban	0.722	0.487	2.201	0.138	2.059	0.793	5.347
	Educational attainment (ref: High school or above)							
	Primary school or below	−1.442	0.836	2.975	0.085	0.236	0.046	1.217
	Junior high or technical secondary school	−0.76	0.771	0.971	0.324	0.467	0.103	2.121
	Monthly household income per capita (¥) (ref: ≥3,001)							
	≤1,000	−0.452	0.88	0.264	0.607	0.636	0.113	3.568
	1,001–3,000	0.79	0.745	1.122	0.289	2.202	0.511	9.49
	Occupational status (ref: Non-retired)							
	Retired	−0.3	0.486	0.382	0.537	0.741	0.286	1.92
	Smoke status(ref: Non-smoker)							
	Smoker	−0.394	0.466	0.715	0.398	0.674	0.271	1.681
	Alcohol consumption (ref: Non-drinker)							
	Drinker	−1.331	0.453	8.634	0.003	0.264	0.109	0.642
	Duration of chronic disease (ref: >10 years)							
	<3 years	−0.973	0.527	3.41	0.065	0.378	0.134	1.062
	3–10 years	−0.828	0.446	3.439	0.064	0.437	0.182	1.048
3	Sex (ref: Female)							
	Male	1.563	0.545	8.23	0.004	4.773	1.641	13.887
	Age(years) (ref: ≥71 years)							
	60–70	1.75	0.483	13.143	<0.001	5.755	2.234	14.823
	Marital status (ref: Divorced/Widowed)							
	Married	−0.699	0.642	1.183	0.277	0.497	0.141	1.752
	Place of residence (ref: Rural)							
	Urban	1.007	0.478	4.446	0.035	2.738	1.074	6.981
	Educational attainment (ref: High school or above)							
	Primary school or below	−4.234	0.998	17.988	<0.001	0.014	0.002	0.103
	Junior high or technical secondary school	−1.888	0.79	5.715	0.017	0.151	0.032	0.712
	Monthly household income per capita (¥) (ref: ≥3,001)							
	≤1,000	−0.08	1.006	0.006	0.937	0.924	0.128	6.638
	1,001–3,000	0.158	0.77	0.042	0.838	1.171	0.259	5.295
	Occupational status (ref: Non-retired)							
	Retired	−0.15	0.568	0.07	0.792	0.861	0.282	2.622
	Smoke status(ref: Non-smoker)							
	Smoker	0.037	0.546	0.005	0.946	1.038	0.356	3.028
	Alcohol consumption (ref: Non-drinker)							
	Drinker	−2.259	0.544	17.258	<0.001	0.104	0.036	0.303
	Duration of chronic disease (ref: >10 years)							
	<3 years	−3.379	0.744	20.638	<0.001	0.034	0.008	0.146
	3–10 years	−1.325	0.493	7.224	0.007	0.266	0.101	0.698

The low literacy-limited information comprehension type served as the reference group. Group 2: moderate literacy-physician-dependent communication type. Group 3: high literacy-autonomous and efficient decision-making type.

Before interpreting the regression results, we checked for multicollinearity. VIF values for all 10 predictors ranged from 1.040 to 1.897, and tolerance values ranged from 0.527 to 0.962, (Supplementary Table 5) indicating no serious multicollinearity (all VIFs < 4.00; all tolerances > 0.25; Supplementary Table 5).

### 3.6 Significant variations in self-management levels were observed among patients with various health literacy profiles and chronic disease comorbidities

After adjusting for age, sex, education, residence, and disease duration, ANCOVA revealed statistically significant differences in self-management behavior scores across the three health literacy profiles. Bonferroni-corrected pairwise comparisons showed that the high literacy-autonomous and efficient decision-making group consistently scored highest across all dimensions. For the total score and medication adherence, all three groups differed significantly from each other (*p* < 0.05). For dietary habits, exercise management, emotional management, and social support, significant differences were observed between the high literacy group and the other two groups (*p* < 0.05), while differences between the low and moderate literacy groups were not statistically significant (*p* > 0.05). As detailed in Table 5.

**Table 5:** Comparison of self-management behavior scores among older adults patients with chronic diseases across different latent profiles of health literacy (adjusted mean ± SE).

Dimension	Low literacy-limited information comprehension (*n* = 56)	Moderate literacy-physician-dependent communication (*n* = 129)	High literacy-autonomous and efficient decision-making (*n* = 117)	F	*p*
Dietary habits	13.025 ± 0.339	13.069 ± 0.205	14.621 ± 0.238	11.798	<0.001
Exercise management	10.599 ± 0.326	11.412 ± 0.197	12.576 ± 0.229	11.322	<0.001
Medication adherence	12.229 ± 0.280	13.672 ± 0.169	15.552 ± 0.197	42.432	<0.001
Emotional management	11.460 ± 0.289	12.149 ± 0.174	13.659 ± 0.203	19.928	<0.001
Social support	14.733 ± 0.265	15.044 ± 0.160	15.695 ± 0.186	4.466	0.012
Total score	62.045 ± 0.924	65.345 ± 0.558	72.102 ± 0.649	39.992	<0.001

Data are presented as adjusted mean ± standard error. Adjusted for age, sex, education, residence, and disease duration as covariates. All F-values were obtained from analysis of covariance (ANCOVA). Bonferroni-corrected pairwise comparisons: see Results section for details.

## 4 Discussion

### 4.1 Overall health literacy levels among older adults patients with chronic disease comorbidities

Health literacy is a crucial protective factor that determines individual health outcomes. The State Council’s Opinions on Implementing the Healthy China Initiative include 15 action-specific indicators related to national health literacy, reflecting an increasing national emphasis on enhancing public health literacy. The overall health literacy score for the 302 patients in this study was (84.37 ± 15.59) points, with an average item score of (3.52 ± 0.65) points. This score indicates a relatively low level of health literacy among the study participants. It should be noted that this classification is relative to the sample distribution rather than an absolute clinical threshold, as the scale lacks externally validated cutoffs. Research indicates that health literacy levels vary across regions (28, 29). In this dataset, 77.5% of patients resided in urban areas, a proportion higher than that in most comparable studies. Compared to rural areas, urban environments offer advantages in terms of economic development, medical facilities, and hospital follow-up systems, which are more conducive to enhancing patients’ health literacy (30). In this study, 69.9% of the patients were retirees. This demographic typically possesses more time and energy to focus on their health status (31), which may partly explain the higher proportion of individuals with high health literacy in this cohort. Notably, the results indicate that 61.2% of participants still fall into the low-to-moderate health literacy group, consistent with previous findings of generally low health literacy among patients with chronic diseases (32).

### 4.2 Heterogeneity in health literacy among older adults patients with chronic disease comorbidities

The analysis of patient health literacy revealed three distinct categories: “low literacy-limited information comprehension,” “moderate literacy-physician-dependent communication,” and “high literacy-autonomous and efficient decision-making.” This highlights the heterogeneity of health literacy among patients with chronic diseases. Data revealed that the low literacy-limited information comprehension group constituted 18.5% of the total sample, scoring lowest across all health literacy items with an overall mean score of 2.58 ± 0.30. This category of older adult patients with chronic disease comorbidities demonstrated overall lower information acquisition and communication abilities than the other two categories, consistent with the findings of Chen et al. (33). This may be attributed to the predominantly rural residence of this population. Given geographical constraints, rural areas typically exhibit lower income, employment, educational opportunities, and healthcare accessibility (34). Consequently, these individuals may fail to recognize the importance of learning health knowledge and resist discussing disease-related topics with others, thereby impairing health literacy among older adults with chronic disease comorbidities (35). The moderate literacy-physician-dependent communication category accounted for 42.6% of the total sample, representing its largest proportion. This may correlate with the province’s overall health literacy levels. Characterized by reliance on physician-patient communication, these patients still require external guidance in health decision-making. Structured education and follow-up support are suitable approaches for enhancing self-management confidence (36). High literacy-autonomous and efficient decision-making group comprised 38.9% of the total sample and achieved the highest average item score (4.17 ± 0.29). This group demonstrated strong autonomous decision-making and information application capabilities, predominantly among urban, highly educated, and high-income individuals. These patients were more likely to have participated in health education programs and acquired medical knowledge, making them suitable “peer supporters” in health education initiatives. Previous studies have shown that peer support delivered via face-to-face interactions, telephone, the Internet, or other emerging technologies (37) aids patients in managing multiple chronic conditions, although research on online platforms remains limited.

Although the three classes show a clear gradient in overall health literacy scores, subtle differences in item-level patterns provide additional descriptive value for understanding class characteristics. Nonetheless, these profiles should be interpreted primarily as reflecting different levels of health literacy within this sample, and the labels are used for descriptive convenience rather than to imply qualitatively distinct categories.

### 4.3 Influencing factors of potential health literacy profiles among older adults patients with chronic disease comorbidities

#### 4.3.1 Impact of sociodemographic factors on health literacy in older adults patients with chronic disease comorbidities

Using the “low literacy-limited information comprehension” class as the reference, our multivariate logistic regression analysis identified six independent predictors of health literacy class membership in multimorbid older adults: sex, age, alcohol consumption, place of residence, education level, and chronic disease duration. Regarding sex, male patients had significantly higher odds than females of being in either the “moderate literacy-physician-dependent communication” class (OR = 4.227; 95% CI: 1.670–10.700; *p* = 0.002) or the “high literacy-autonomous and efficient decision-making” class (OR = 4.773; 95% CI: 1.641–13.887; *p* = 0.004). This finding agrees with Xie’s results (38). Previous studies on sex differences in health literacy have been inconsistent. Some evidence indicates that women may have better health literacy, possibly because they take on more family caregiving duties and use healthcare services more often (39). However, most published work suggests that older men tend to have higher health literacy than older women (19). This inconsistency might reflect a greater tendency among men—especially those with multimorbidity—to take an information-driven approach when making health-related choices.

With respect to age, participants aged 60–70 years were significantly more likely than those over 70 to belong to the higher-literacy classes—specifically, the “moderate literacy–physician-dependent communication” class (OR = 2.364; 95% CI: 1.037–5.389; *p* = 0.041) and the “high literacy–autonomous and efficient decision-making” class (OR = 5.755; 95% CI: 2.234–14.823; *p* < 0.001). These results confirm age as an important sociodemographic correlate of health literacy, matching earlier findings by Liu et al. (40). A reasonable explanation is that age-related declines in cognitive functions—such as comprehension, problem-solving, information processing, and memory—may reduce older adults’ ability to follow health-related instructions and to find and use relevant health information and resources (41). Concerning place of residence, urban residents had significantly higher health literacy than their rural counterparts (OR = 2.738; 95% CI: 1.074–6.981; *p* = 0.035), a pattern consistent with Mohammad et al.’s report (42). This gap may be partly explained by China’s household registration (hukou) system, which creates unequal access to social welfare and healthcare. Rural residents often encounter greater obstacles in obtaining high-quality medical care, joining preventive or digital health services, and engaging in proactive health-seeking. In addition, primary care facilities in rural areas tend to have weaker infrastructure, fewer staff, and less service capacity. Beauchamp et al. (43) did not find a significant urban–rural health literacy difference in their Australian sample, but the urban–rural gap in educational quality is much smaller in Australia than in China—a key contextual factor that likely explains the differing results. Our findings also match a recent study on healthcare-seeking behavior among older adults in Fujian Province, China (44).

As for education level, patients with primary school education or less (OR = 0.014; 95% CI: 0.002–0.103; *p* < 0.001) and those with junior high or vocational school education (OR = 0.151; 95% CI: 0.032–0.712; *p* = 0.017) were significantly more likely to fall into the “low literacy–limited information comprehension” class. This aligns with Yang et al. (45). Less educated individuals face multiple interconnected challenges: they have more trouble communicating disease-related information with healthcare providers (46), encounter structural and systemic barriers to timely and appropriate care (47), and have limited ability to draw on social and informational resources (19). All of these factors restrict their capacity to obtain, understand, and use relevant health information. In contrast, older adults with more education generally show stronger skills in acquiring, interpreting, and critically evaluating health information. They also tend to have more flexible time to focus on their own health and are better at using digital tools—such as online health platforms, reputable medical websites, and mobile apps—to access evidence-based knowledge on medication, nutrition, and chronic disease management. These advantages together contribute to better health-related problem-solving and more effective communication with healthcare providers (48). Notably, marital status was significantly associated with health literacy class membership in the univariate analysis but lost statistical significance in the multivariate model. This attenuation was not due to multicollinearity, as confirmed by the variance inflation factor (VIF) for marital status (VIF = 1.164), which falls well below the conventional threshold of 4.00. Instead, the loss of significance likely reflects confounding by age and living arrangement—two variables strongly correlated with marital status among older adults. In our sample, married participants were, on average, younger and more likely to live with family members—factors independently linked to higher health literacy levels. Consequently, the observed association between marital status and health literacy may be partially mediated by age-related social support structures. This underscores the necessity of multivariate adjustment when evaluating the independent contribution of sociodemographic factors to health literacy profiles.

#### 4.3.2 Impact of disease-related factors on health literacy among older adults patients with chronic disease comorbidities

This study also examined the association between chronic disease duration and health literacy. Results indicated that individuals with a disease duration of less than 3 years (OR = 0.034, 95% CI: 0.008–0.146, *p* < 0.001) and those with a duration of 3–10 years (OR = 0.266, 95% CI: 0.101–0.698, *p* = 0.007) were significantly more likely to be classified in the “low literacy–limited information comprehension” profile. In other words, compared with patients having shorter disease durations, those with a disease duration exceeding 10 years exhibited a substantially lower likelihood of belonging to this low-skill profile—suggesting comparatively higher health literacy levels. This finding aligns with the results reported by Lu et al.^[50]^and is consistent with Chen et al. (20), both of whom observed a positive association between longer disease duration and enhanced health literacy. Notably, longer disease duration was also associated with greater self-efficacy. Several interpretations of this association are plausible. One mechanism is that extended disease duration affords patients repeated opportunities—through routine clinical encounters, ongoing self-monitoring, and longitudinal disease management—to access, practice, and internalize health-related knowledge. As patients accumulate successful self-management experiences, their confidence in managing their condition may increase, thereby fostering improvements in health literacy over time. However, an equally plausible interpretation is that individuals with higher health literacy are better equipped to manage their chronic conditions, adhere to complex treatment regimens, and effectively navigate the healthcare system, thereby extending their survival with multimorbidity. These two explanations are not mutually exclusive, but the cross-sectional design of the present study precludes any definitive conclusions regarding the direction of causality. Future longitudinal studies are needed to clarify the temporal and causal relationships between disease duration and health literacy. Notwithstanding this caveat, the high statistical significance of these associations (*p* < 0.001) underscores the robustness of the observed relationship. Taken together, these findings suggest that disease duration is a clinically meaningful indicator that may guide the timing and intensity of health literacy interventions.

#### 4.3.3 Differences in self-management behaviors among older adults patients with chronic disease comorbidities across potential health literacy categories

The unpredictability of chronic diseases in older adults underscores the critical importance of self-management behavior in disease control. Although effectiveness may vary by disease type, patient characteristics, and environment, effective self-management is essential for managing any chronic condition (49). Sustained adherence to self-management tasks, such as dietary control, medication management, and symptom monitoring, is pivotal for health outcomes but poses significant challenges for patients. Our findings revealed significant differences in self-management behaviors among older adult patients with chronic disease comorbidity across distinct health literacy categories after controlling for age, sex, education, residence, and disease duration (*F* = 39.992, *p* < 0.001, partial η^2^ = 0.214). Patients classified as having low literacy-limited information comprehension type demonstrated the lowest self-management scores, whereas those categorized as having high literacy with efficient autonomous decision-making achieved the highest scores. Previous research (50) consistently shows that health literacy is significantly associated with self-management behaviors among patients with chronic diseases. For dietary habits, exercise management, and social support, the low and moderate literacy groups showed similar levels, both significantly lower than the high literacy group. However, for medication adherence and total score, all three groups differed significantly from each other, with the high literacy group consistently achieving the highest scores. Specifically, in this study, patients with low literacy-limited information comprehension, as well as those with moderate literacy-physician-dependent communication, exhibited poorer medication adherence. Their advanced age, lower educational attainment, diminished cognitive abilities, and limited participation in health education contributed to their low health literacy and suboptimal self-management behaviors, consistent with the findings of Sankhi et al. (51). Conversely, patients with high literacy-autonomous and efficient decision-making type demonstrated superior capabilities owing to their higher education and socioeconomic status. This enables them to effectively obtain, evaluate, and make informed decisions based on health information, thereby prioritizing medication adherence after discharge. This is consistent with the conclusions drawn by Shan et al. (52). Additionally, while there was no difference in emotional management between low literacy-limited information comprehension patients and moderate literacy-physician-dependent communication patients, both groups demonstrated significantly poorer emotional management than high literacy-autonomous and efficient decision-making patients. A possible explanation is that chronic disease comorbidity compromises patients’ physical function, severely impacting the mental health of older adults. Older adult patients with low health literacy exhibit poor self-management capabilities, and the prolonged absence of improvement coupled with high recurrence rates makes them more susceptible to anxiety and depression (53). Research indicates that the chronic nature of these conditions, coupled with low cure rates and high disability rates, leads to deteriorating physical health among older adults (54). This diminishes their confidence in coping with disease-related negative impacts, making them more susceptible to severe psychological problems. Therefore, in clinical practice, healthcare providers should consider tailored interventions based on patients’ health literacy profiles. For the low literacy group, priority should be given to enhancing coping abilities and emotional support. For the moderate literacy group, strengthening medication adherence and self-management skills may be particularly beneficial. For the high literacy group, strategies to maintain their autonomous decision-making capacity should be reinforced.

### 4.4 Limitations

This study’s valuable findings may be impacted by limitations that could influence its internal and external validity. When interpreting the results and planning future research, it is essential to consider the following: Firstly, as this was a cross-sectional study, it can only demonstrate associations among variables and not establish causal relationships between health literacy, its determinants, and self-management behaviors. It is unclear whether low health literacy causes poor self-management behaviors or if inadequate self-management over the long term impedes improvements in health literacy. To confirm causal pathways, longitudinal or interventional studies are necessary. Secondly, the study utilized convenience sampling, recruiting all participants from a single tertiary-level hospital in Liaoning Province. While this approach ensured homogeneity within a specific context, it restricted the generalizability of the findings to broader populations, such as community-dwelling older adults patients, individuals in regions with varying economic development levels, or patients from other healthcare facilities. Future research should expand the sampling scope and conduct multicenter investigations to enhance representativeness. Additionally, the exclusion of patients with severe visual, hearing, or mental impairments may have systematically excluded older adults with the lowest health literacy, potentially leading to an overestimation of health literacy levels in our sample. Furthermore, although family members were only permitted to serve as scribes—recording answers dictated by the patients themselves, with the patient present throughout—this practice may still have introduced social desirability bias, as patients might have felt hesitant to express their true understanding or concerns in front of their relatives. These factors should be considered when interpreting the findings. Thirdly, the “classify-then-analyze” approach used in this study does not account for classification uncertainty. However, the excellent classification quality (entropy = 0.941; average posterior probabilities > 0.96) suggests that any potential bias is likely minimal (55). Fourthly, although the Health Literacy Measurement Scale used in this study has been translated into Chinese and validated, its dimensional structure may not fully align with the unique context of Chinese older adults with chronic disease comorbidity (e.g., understanding of polypharmacy safety and awareness of comorbidity co-management). Further cultural adaptation and validation are required. Similarly, the Self-Management Behavior Scale may not comprehensively capture the complex behaviors that are specific to comorbidity management. Fifth, although the local independence assumption was reasonably met, minor residual dependencies were observed among a few items within the same subscales, suggesting potential item redundancy that may have had a minor influence on the class structure. However, the high entropy (0.941) and satisfactory classification probabilities suggest that any such dependence did not materially alter the main conclusions. Finally, there were sample size constraints on subgroup analysis. Although the total sample size met the requirements for this study, the sample sizes in certain subgroups (e.g., low literacy-limited information comprehension, *n* = 56) were relatively small. Additionally for participants with elementary school education or below (OR = 0.014, 95% CI: 0.002–0.103), the wide confidence interval is primarily attributable to the limited sample size in this subtype. These results should be interpreted with caution, with emphasis on the direction and clinical significance of the effects rather than the exact numerical values. Future studies with larger and more balanced samples are needed to obtain more stable and precise estimates.

Through latent profile analysis of health literacy among older adults patients with chronic disease comorbidity, this study identified three distinct categories: low literacy-limited information comprehension, moderate literacy-physician-dependent communication, and high literacy-autonomous and efficient decision-making. Further findings revealed structural differences across self-management behavior dimensions among these categories, indicating that health literacy disparities are significantly associated with patients’ self-management execution capacity and behavioral patterns. The identified profiles represent relative groupings within this sample rather than externally validated clinical categories.

These findings have important implications for clinical practice and health policy, particularly within the context of China’s tiered healthcare system and the National Basic Public Health Service Program.

First, community health centers could incorporate a brief health literacy screening into the annual health examination for older adults—a service already mandated by the national older adults health management program. Based on the screening results, patients could be classified into one of the three profiles, enabling targeted health education and management plans. This operationalizes the policy requirement for “classified and graded health management” of chronic disease patients. Second, health education resources should be differentiated according to patient characteristics. For the low literacy-limited information comprehension group, face-to-face education with visual aids (e.g., pictorial medication instructions, simplified dietary charts) should be prioritized during routine follow-up visits, focusing on foundational disease knowledge and basic self-care skills. For the moderate literacy-physician-dependent communication group, interventions should strengthen physician-patient communication through structured health education integrated into each follow-up contact, leveraging the family doctor contracting service model. For the high literacy-autonomous decision-making group, digital health tools (e.g., patient portals, WeChat-based platforms) and peer support groups can be employed to support autonomous self-management. Third, the implementation of such stratified interventions should align with the Guidelines on Strengthening Primary Chronic Disease Health Management Services (2025), which emphasizes integrated prevention, screening, diagnosis, treatment, management, and rehabilitation services. Digital health technologies should also be leveraged to ensure equitable access to literacy support, particularly for underserved populations. Future research should examine the long-term effectiveness of such stratified interventions to further refine the evidence base for precision chronic disease management.

## Data Availability

The original contributions presented in the study are included in the article/Supplementary material, further inquiries can be directed to the corresponding author.
