# Correction: The latent profile of health literacy among older adults patients with chronic disease comorbidity and its association with self-management behaviors

**DOI:** 10.3389/fpubh.2026.2007038

**Published:** 2026-10-05

**Authors:** Jianrui Guo, Bo Ma, Wenjing Xue

**Affiliations:** 1 Jinzhou Medical University, Jinzhou, China; 2 Liaoning Provincial People's Hospital, Shenyang, China

**Keywords:** chronic disease, health literacy, latent profile analysis, older adults, self-management behaviors

The tables were in the wrong order in the published version of this paper. The previous Table 1 has been renumbered as Table 5. The corrected table order is as follows:

Table 1. Model fit indices for latent profile analysis of health literacy among older adults patients with chronic disease comorbidities (*n* = 302).

Table 2. Univariate analysis of latent profiles of health literacy among older adults patients with chronic disease comorbidities.

Table 3. Variable assignment.

Table 4. Multivariate logistic regression analysis of health literacy profiles among older adults patients with chronic disease comorbidities (*n* = 302).

Table 5. Comparison of self-management behavior scores among older adults patients with chronic diseases across different latent profiles of health literacy (adjusted mean ± SE).

All corresponding in-text table citations have been updated accordingly.

The order has now been corrected.

The original article contained incorrect table citations and a missing sentence in Section 3.5.

A correction has been made to the section Section 3.5 (Multivariate analysis of latent categories of health literacy among older adults patients with chronic disease comorbidities):

The sentence “Dummy coding was applied to categorical variables; the coding scheme is presented in Table 4.” has been changed to:

“Dummy coding was applied to categorical variables; the coding scheme is presented in Table 3.”

After this sentence, the following sentence has been added:

“The results of the multivariate analysis are presented in Table 4.”

In the sentence “(all VIFs < 4.00; all tolerances > 0.25; Table 5)”, “Table 5” has been changed to “Supplementary Table 5.”

The original version of this article has been updated.

