# Supplementary material for: The latent profile of health literacy among older adults patients with chronic disease comorbidity and its association with self-management behaviors
Source: Front Public Health. 2026 Sep 11;14:1880321. doi: 10.3389/fpubh.2026.1880321 (PMC13612451; doi:10.3389/fpubh.2026.1880321)
Supplement: Supplementary file 1 [file Table_1.DOCX]

Supplementary Table 1.The complete Cohen's d values for all indicators across adjacent classes

| Indicator | Class 1 vs Class 2 | Class 2 vs Class 3 |
| --- | --- | --- |
| A1 | 0.77 | 1.52 |
| A2 | 0.89 | 0.74 |
| A3 | 0.87 | 1.60 |
| A4 | 0.80 | 0.62 |
| A5 | 0.87 | 0.27 |
| A6 | 0.70 | 1.60 |
| A7 | 0.94 | 0.92 |
| A8 | 1.18 | 1.31 |
| A9 | 0.63 | 1.45 |
| A10 | 1.12 | 1.37 |
| A11 | 0.71 | 0.86 |
| A12 | 1.20 | 0.53 |
| A13 | 0.45 | 0.11 |
| A14 | 0.47 | 0.55 |
| A15 | 0.44 | 0.85 |
| A16 | 0.33 | 0.50 |
| A17 | 0.63 | 0.75 |
| A18 | 0.81 | 0.66 |
| A19 | 0.91 | 1.02 |
| A20 | 0.50 | 0.33 |
| A21 | 0.79 | 0.59 |
| A22 | 0.74 | 0.59 |
| A23 | 0.75 | 0.78 |
| A24 | 0.61 | 0.65 |

| Variable | Category | Frequency (n) | Percentage (%) |
| --- | --- | --- | --- |
| Age (years) | 60～70 | 171 | 56.6 |
|  | ≥71 | 131 | 43.4 |
| Sex | Male | 184 | 60.9 |
|  | Female | 118 | 39.1 |
| Marital status | Married | 253 | 83.8 |
|  | Divorced/Widowed | 49 | 16.2 |
| Place of residence | Urban | 234 | 77.5 |
|  | Rural | 68 | 22.5 |
| Educational attainment | Primary school or below | 70 | 23.2 |
|  | Junior high or technical secondary school | 146 | 48.3 |
|  | High school or above | 86 | 28.5 |
| Monthly household income per capita (¥) | ≤1000 | 48 | 15.9 |
|  | 1001～3000 | 188 | 62.3 |
|  | ≥3001 | 66 | 21.9 |
| Occupational status | Retired | 211 | 69.9 |
|  | Non-retired | 91 | 30.1 |
| Number of children | One | 194 | 64.2 |
|  | Two or more | 108 | 35.8 |
| Smoking status | Smoker | 89 | 29.5 |
|  | Non-smoker | 213 | 70.5 |
| Alcohol consumption | Drinker | 101 | 33.4 |
|  | Non-drinker | 201 | 66.6 |
| Duration of chronic disease (years) | Less than 3 years | 48 | 15.9 |
|  | 3 to 10 years | 117 | 38.7 |
|  | More than 10 years | 137 | 45.4 |
| Payment method | Employee medical insurance | 165 | 54.6 |
|  | Others | 137 | 45.4 |
| BMI | ＜24 | 138 | 45.7 |
|  | 24～28 | 124 | 41.1 |
|  | ＞28 | 40 | 13.2 |

Supplementary Table 2. Sociodemographic and clinical characteristics of the study participants(N=302)

Supplementary Table 3. Item-level discrimination of the 24 health literacy items across three latent classes

| Item | Class 1 (n=56) | Class 2 (n=129) | Class 3 (n=117) | F | *P* |
| --- | --- | --- | --- | --- | --- |
| A1 | 1.84 ± 0.63 | 2.61 ± 0.76 | 4.13 ± 0.84 | 204.78 | < 0.001 |
| A2 | 3.09 ± 0.51 | 3.98 ± 0.54 | 4.72 ± 0.47 | 201.16 | < 0.001 |
| A3 | 2.04 ± 0.66 | 2.91 ± 0.76 | 4.51 ± 0.68 | 276.06 | < 0.001 |
| A4 | 3.14 ± 0.52 | 3.94 ± 0.57 | 4.56 ± 0.56 | 124.22 | < 0.001 |
| A5 | 3.77 ± 0.71 | 4.64 ± 0.53 | 4.91 ± 0.29 | 101.41 | < 0.001 |
| A6 | 1.32 ± 0.69 | 2.02 ± 0.97 | 3.62 ± 1.05 | 138.75 | < 0.001 |
| A7 | 2.79 ± 0.68 | 3.73 ± 0.73 | 4.65 ± 0.55 | 162.93 | < 0.001 |
| A8 | 1.95 ± 1.14 | 3.13 ± 1.25 | 4.44 ± 0.81 | 110.07 | < 0.001 |
| A9 | 1.41 ± 0.57 | 2.04 ± 0.85 | 3.49 ± 0.82 | 165.63 | < 0.001 |
| A10 | 1.84 ± 1.01 | 2.96 ± 1.25 | 4.33 ± 0.90 | 111.22 | < 0.001 |
| A11 | 2.82 ± 0.58 | 3.53 ± 0.71 | 4.39 ± 0.66 | 115.99 | < 0.001 |
| A12 | 2.89 ± 0.85 | 4.09 ± 0.71 | 4.62 ± 0.55 | 122.21 | < 0.001 |
| A13 | 4.14 ± 0.84 | 4.59 ± 0.61 | 4.70 ± 0.51 | 15.57 | < 0.001 |
| A14 | 1.30 ± 0.57 | 1.77 ± 0.84 | 2.32 ± 0.94 | 30.75 | < 0.001 |
| A15 | 1.27 ± 0.65 | 1.71 ± 0.98 | 2.56 ± 1.30 | 33.47 | < 0.001 |
| A16 | 1.79 ± 0.71 | 2.12 ± 0.76 | 2.62 ± 0.94 | 22.16 | < 0.001 |
| A17 | 2.91 ± 0.61 | 3.54 ± 0.85 | 4.29 ± 0.72 | 68.24 | < 0.001 |
| A18 | 3.23 ± 0.87 | 4.04 ± 0.82 | 4.70 ± 0.55 | 77.14 | < 0.001 |
| A19 | 2.57 ± 1.01 | 3.48 ± 1.04 | 4.50 ± 0.76 | 86.79 | < 0.001 |
| A20 | 3.84 ± 0.93 | 4.34 ± 0.77 | 4.67 ± 0.59 | 24.07 | < 0.001 |
| A21 | 3.23 ± 1.01 | 4.02 ± 0.93 | 4.61 ± 0.69 | 48.97 | < 0.001 |
| A22 | 2.96 ± 0.97 | 3.70 ± 1.06 | 4.29 ± 0.88 | 35.95 | < 0.001 |
| A23 | 2.70 ± 0.74 | 3.45 ± 0.98 | 4.23 ± 0.83 | 60.65 | < 0.001 |
| A24 | 2.96 ± 0.71 | 3.57 ± 0.86 | 4.22 ± 0.81 | 48.58 | < 0.001 |

Notes: Data are presented as mean ± standard deviation. Class 1 = low literacy–limited information comprehension type; Class 2 = moderate literacy–physician-dependent communication type; Class 3 = high literacy–autonomous and efficient decision-making type. All P-values were obtained from one-way ANOVA. All 24 items showed statistically significant differences across the three classes ( P < 0.001)

Values are presented as mean ± standard deviation. These descriptive comparisons are presented for illustrative purposes; the latent classes were derived from these indicators, so between‑class differences are expected by design and do not constitute independent validation.

Supplementary Table 4. Pearson correlation matrix among the 24 items of the Health Literacy Measurement Scale for Patients with Chronic Diseases

|  | |  | A2 | A3 | A4 | A5 | A6 | A7 | A8 | A9 | A10 | A11 | A12 | A13 | A14 | A15 | A16 | A17 | A18 | A19 | A20 | A21 | A22 | A23 | A24 |
| --- | --- | --- | --- | --- | --- | --- | --- | --- | --- | --- | --- | --- | --- | --- | --- | --- | --- | --- | --- | --- | --- | --- | --- | --- | --- |
| A1 | Pearson Correlation | 1 | .661** | .912** | .599** | .431** | .565** | .504** | .682** | .593** | .695** | .511** | .491** | .110 | .293** | .272** | .297** | .350** | .309** | .387** | .244** | .287** | .248** | .367** | .452** |
|  | Sig. (2-tailed) |  | .000 | .000 | .000 | .000 | .000 | .000 | .000 | .000 | .000 | .000 | .000 | .055 | .000 | .000 | .000 | .000 | .000 | .000 | .000 | .000 | .000 | .000 | .000 |
|  | N | 302 | 302 | 302 | 302 | 302 | 302 | 302 | 302 | 302 | 302 | 302 | 302 | 302 | 302 | 302 | 302 | 302 | 302 | 302 | 302 | 302 | 302 | 302 | 302 |
| A2 | Pearson Correlation | .661** | 1 | .691** | .604** | .485** | .461** | .590** | .531** | .559** | .526** | .572** | .570** | .233** | .332** | .362** | .315** | .434** | .485** | .519** | .258** | .367** | .321** | .396** | .412** |
|  | Sig. (2-tailed) | .000 |  | .000 | .000 | .000 | .000 | .000 | .000 | .000 | .000 | .000 | .000 | .000 | .000 | .000 | .000 | .000 | .000 | .000 | .000 | .000 | .000 | .000 | .000 |
|  | N | 302 | 302 | 302 | 302 | 302 | 302 | 302 | 302 | 302 | 302 | 302 | 302 | 302 | 302 | 302 | 302 | 302 | 302 | 302 | 302 | 302 | 302 | 302 | 302 |
| A3 | Pearson Correlation | .912** | .691** | 1 | .596** | .428** | .611** | .576** | .688** | .640** | .708** | .565** | .524** | .134* | .401** | .347** | .301** | .358** | .400** | .460** | .274** | .329** | .252** | .381** | .427** |
|  | Sig. (2-tailed) | .000 | .000 |  | .000 | .000 | .000 | .000 | .000 | .000 | .000 | .000 | .000 | .020 | .000 | .000 | .000 | .000 | .000 | .000 | .000 | .000 | .000 | .000 | .000 |
|  | N | 302 | 302 | 302 | 302 | 302 | 302 | 302 | 302 | 302 | 302 | 302 | 302 | 302 | 302 | 302 | 302 | 302 | 302 | 302 | 302 | 302 | 302 | 302 | 302 |
| A4 | Pearson Correlation | .599** | .604** | .596** | 1 | .474** | .477** | .604** | .447** | .484** | .436** | .496** | .512** | .216** | .336** | .271** | .225** | .456** | .422** | .458** | .193** | .347** | .296** | .339** | .376** |
|  | Sig. (2-tailed) | .000 | .000 | .000 |  | .000 | .000 | .000 | .000 | .000 | .000 | .000 | .000 | .000 | .000 | .000 | .000 | .000 | .000 | .000 | .001 | .000 | .000 | .000 | .000 |
|  | N | 302 | 302 | 302 | 302 | 302 | 302 | 302 | 302 | 302 | 302 | 302 | 302 | 302 | 302 | 302 | 302 | 302 | 302 | 302 | 302 | 302 | 302 | 302 | 302 |
| A5 | Pearson Correlation | .431** | .485** | .428** | .474** | 1 | .343** | .478** | .433** | .353** | .460** | .374** | .415** | .177** | .249** | .263** | .208** | .353** | .288** | .367** | .329** | .282** | .252** | .313** | .377** |
|  | Sig. (2-tailed) | .000 | .000 | .000 | .000 |  | .000 | .000 | .000 | .000 | .000 | .000 | .000 | .002 | .000 | .000 | .000 | .000 | .000 | .000 | .000 | .000 | .000 | .000 | .000 |
|  | N | 302 | 302 | 302 | 302 | 302 | 302 | 302 | 302 | 302 | 302 | 302 | 302 | 302 | 302 | 302 | 302 | 302 | 302 | 302 | 302 | 302 | 302 | 302 | 302 |
| A6 | Pearson Correlation | .565** | .461** | .611** | .477** | .343** | 1 | .641** | .524** | .693** | .515** | .545** | .420** | .084 | .376** | .391** | .221** | .392** | .396** | .434** | .180** | .336** | .320** | .369** | .354** |
|  | Sig. (2-tailed) | .000 | .000 | .000 | .000 | .000 |  | .000 | .000 | .000 | .000 | .000 | .000 | .148 | .000 | .000 | .000 | .000 | .000 | .000 | .002 | .000 | .000 | .000 | .000 |
|  | N | 302 | 302 | 302 | 302 | 302 | 302 | 302 | 302 | 302 | 302 | 302 | 302 | 302 | 302 | 302 | 302 | 302 | 302 | 302 | 302 | 302 | 302 | 302 | 302 |
| A7 | Pearson Correlation | .504** | .590** | .576** | .604** | .478** | .641** | 1 | .498** | .573** | .471** | .623** | .555** | .231** | .343** | .336** | .232** | .515** | .568** | .567** | .281** | .427** | .389** | .430** | .360** |
|  | Sig. (2-tailed) | .000 | .000 | .000 | .000 | .000 | .000 |  | .000 | .000 | .000 | .000 | .000 | .000 | .000 | .000 | .000 | .000 | .000 | .000 | .000 | .000 | .000 | .000 | .000 |
|  | N | 302 | 302 | 302 | 302 | 302 | 302 | 302 | 302 | 302 | 302 | 302 | 302 | 302 | 302 | 302 | 302 | 302 | 302 | 302 | 302 | 302 | 302 | 302 | 302 |
| A8 | Pearson Correlation | .682** | .531** | .688** | .447** | .433** | .524** | .498** | 1 | .520** | .931** | .523** | .472** | .022 | .251** | .197** | .202** | .209** | .351** | .493** | .221** | .142* | .090 | .229** | .271** |
|  | Sig. (2-tailed) | .000 | .000 | .000 | .000 | .000 | .000 | .000 |  | .000 | .000 | .000 | .000 | .700 | .000 | .001 | .000 | .000 | .000 | .000 | .000 | .013 | .119 | .000 | .000 |
|  | N | 302 | 302 | 302 | 302 | 302 | 302 | 302 | 302 | 302 | 302 | 302 | 302 | 302 | 302 | 302 | 302 | 302 | 302 | 302 | 302 | 302 | 302 | 302 | 302 |
| A9 | Pearson Correlation | .593** | .559** | .640** | .484** | .353** | .693** | .573** | .520** | 1 | .531** | .586** | .454** | .145* | .391** | .479** | .346** | .439** | .418** | .431** | .232** | .412** | .375** | .442** | .420** |
|  | Sig. (2-tailed) | .000 | .000 | .000 | .000 | .000 | .000 | .000 | .000 |  | .000 | .000 | .000 | .012 | .000 | .000 | .000 | .000 | .000 | .000 | .000 | .000 | .000 | .000 | .000 |
|  | N | 302 | 302 | 302 | 302 | 302 | 302 | 302 | 302 | 302 | 302 | 302 | 302 | 302 | 302 | 302 | 302 | 302 | 302 | 302 | 302 | 302 | 302 | 302 | 302 |
| A10 | Pearson Correlation | .695** | .526** | .708** | .436** | .460** | .515** | .471** | .931** | .531** | 1 | .500** | .492** | .028 | .264** | .208** | .225** | .231** | .357** | .488** | .239** | .137* | .073 | .224** | .288** |
|  | Sig. (2-tailed) | .000 | .000 | .000 | .000 | .000 | .000 | .000 | .000 | .000 |  | .000 | .000 | .632 | .000 | .000 | .000 | .000 | .000 | .000 | .000 | .017 | .205 | .000 | .000 |
|  | N | 302 | 302 | 302 | 302 | 302 | 302 | 302 | 302 | 302 | 302 | 302 | 302 | 302 | 302 | 302 | 302 | 302 | 302 | 302 | 302 | 302 | 302 | 302 | 302 |
| A11 | Pearson Correlation | .511** | .572** | .565** | .496** | .374** | .545** | .623** | .523** | .586** | .500** | 1 | .488** | .201** | .351** | .363** | .291** | .466** | .471** | .477** | .228** | .376** | .292** | .370** | .339** |
|  | Sig. (2-tailed) | .000 | .000 | .000 | .000 | .000 | .000 | .000 | .000 | .000 | .000 |  | .000 | .000 | .000 | .000 | .000 | .000 | .000 | .000 | .000 | .000 | .000 | .000 | .000 |
|  | N | 302 | 302 | 302 | 302 | 302 | 302 | 302 | 302 | 302 | 302 | 302 | 302 | 302 | 302 | 302 | 302 | 302 | 302 | 302 | 302 | 302 | 302 | 302 | 302 |
| A12 | Pearson Correlation | .491** | .570** | .524** | .512** | .415** | .420** | .555** | .472** | .454** | .492** | .488** | 1 | .305** | .246** | .256** | .221** | .438** | .535** | .513** | .219** | .361** | .293** | .345** | .328** |
|  | Sig. (2-tailed) | .000 | .000 | .000 | .000 | .000 | .000 | .000 | .000 | .000 | .000 | .000 |  | .000 | .000 | .000 | .000 | .000 | .000 | .000 | .000 | .000 | .000 | .000 | .000 |
|  | N | 302 | 302 | 302 | 302 | 302 | 302 | 302 | 302 | 302 | 302 | 302 | 302 | 302 | 302 | 302 | 302 | 302 | 302 | 302 | 302 | 302 | 302 | 302 | 302 |
| A13 | Pearson Correlation | .110 | .233** | .134* | .216** | .177** | .084 | .231** | .022 | .145* | .028 | .201** | .305** | 1 | .260** | .260** | .224** | .340** | .245** | .121* | .352** | .410** | .350** | .361** | .288** |
|  | Sig. (2-tailed) | .055 | .000 | .020 | .000 | .002 | .148 | .000 | .700 | .012 | .632 | .000 | .000 |  | .000 | .000 | .000 | .000 | .000 | .035 | .000 | .000 | .000 | .000 | .000 |
|  | N | 302 | 302 | 302 | 302 | 302 | 302 | 302 | 302 | 302 | 302 | 302 | 302 | 302 | 302 | 302 | 302 | 302 | 302 | 302 | 302 | 302 | 302 | 302 | 302 |
| A14 | Pearson Correlation | .293** | .332** | .401** | .336** | .249** | .376** | .343** | .251** | .391** | .264** | .351** | .246** | .260** | 1 | .565** | .357** | .389** | .281** | .228** | .245** | .300** | .232** | .283** | .291** |
|  | Sig. (2-tailed) | .000 | .000 | .000 | .000 | .000 | .000 | .000 | .000 | .000 | .000 | .000 | .000 | .000 |  | .000 | .000 | .000 | .000 | .000 | .000 | .000 | .000 | .000 | .000 |
|  | N | 302 | 302 | 302 | 302 | 302 | 302 | 302 | 302 | 302 | 302 | 302 | 302 | 302 | 302 | 302 | 302 | 302 | 302 | 302 | 302 | 302 | 302 | 302 | 302 |
| A15 | Pearson Correlation | .272** | .362** | .347** | .271** | .263** | .391** | .336** | .197** | .479** | .208** | .363** | .256** | .260** | .565** | 1 | .390** | .480** | .277** | .263** | .263** | .403** | .329** | .367** | .301** |
|  | Sig. (2-tailed) | .000 | .000 | .000 | .000 | .000 | .000 | .000 | .001 | .000 | .000 | .000 | .000 | .000 | .000 |  | .000 | .000 | .000 | .000 | .000 | .000 | .000 | .000 | .000 |
|  | N | 302 | 302 | 302 | 302 | 302 | 302 | 302 | 302 | 302 | 302 | 302 | 302 | 302 | 302 | 302 | 302 | 302 | 302 | 302 | 302 | 302 | 302 | 302 | 302 |
| A16 | Pearson Correlation | .297** | .315** | .301** | .225** | .208** | .221** | .232** | .202** | .346** | .225** | .291** | .221** | .224** | .357** | .390** | 1 | .411** | .234** | .146* | .224** | .306** | .253** | .217** | .294** |
|  | Sig. (2-tailed) | .000 | .000 | .000 | .000 | .000 | .000 | .000 | .000 | .000 | .000 | .000 | .000 | .000 | .000 | .000 |  | .000 | .000 | .011 | .000 | .000 | .000 | .000 | .000 |
|  | N | 302 | 302 | 302 | 302 | 302 | 302 | 302 | 302 | 302 | 302 | 302 | 302 | 302 | 302 | 302 | 302 | 302 | 302 | 302 | 302 | 302 | 302 | 302 | 302 |
| A17 | Pearson Correlation | .350** | .434** | .358** | .456** | .353** | .392** | .515** | .209** | .439** | .231** | .466** | .438** | .340** | .389** | .480** | .411** | 1 | .483** | .356** | .262** | .505** | .513** | .485** | .406** |
|  | Sig. (2-tailed) | .000 | .000 | .000 | .000 | .000 | .000 | .000 | .000 | .000 | .000 | .000 | .000 | .000 | .000 | .000 | .000 |  | .000 | .000 | .000 | .000 | .000 | .000 | .000 |
|  | N | 302 | 302 | 302 | 302 | 302 | 302 | 302 | 302 | 302 | 302 | 302 | 302 | 302 | 302 | 302 | 302 | 302 | 302 | 302 | 302 | 302 | 302 | 302 | 302 |
| A18 | Pearson Correlation | .309** | .485** | .400** | .422** | .288** | .396** | .568** | .351** | .418** | .357** | .471** | .535** | .245** | .281** | .277** | .234** | .483** | 1 | .719** | .285** | .393** | .322** | .357** | .285** |
|  | Sig. (2-tailed) | .000 | .000 | .000 | .000 | .000 | .000 | .000 | .000 | .000 | .000 | .000 | .000 | .000 | .000 | .000 | .000 | .000 |  | .000 | .000 | .000 | .000 | .000 | .000 |
|  | N | 302 | 302 | 302 | 302 | 302 | 302 | 302 | 302 | 302 | 302 | 302 | 302 | 302 | 302 | 302 | 302 | 302 | 302 | 302 | 302 | 302 | 302 | 302 | 302 |
| A19 | Pearson Correlation | .387** | .519** | .460** | .458** | .367** | .434** | .567** | .493** | .431** | .488** | .477** | .513** | .121* | .228** | .263** | .146* | .356** | .719** | 1 | .159** | .287** | .205** | .267** | .226** |
|  | Sig. (2-tailed) | .000 | .000 | .000 | .000 | .000 | .000 | .000 | .000 | .000 | .000 | .000 | .000 | .035 | .000 | .000 | .011 | .000 | .000 |  | .006 | .000 | .000 | .000 | .000 |
|  | N | 302 | 302 | 302 | 302 | 302 | 302 | 302 | 302 | 302 | 302 | 302 | 302 | 302 | 302 | 302 | 302 | 302 | 302 | 302 | 302 | 302 | 302 | 302 | 302 |
| A20 | Pearson Correlation | .244** | .258** | .274** | .193** | .329** | .180** | .281** | .221** | .232** | .239** | .228** | .219** | .352** | .245** | .263** | .224** | .262** | .285** | .159** | 1 | .361** | .277** | .300** | .232** |
|  | Sig. (2-tailed) | .000 | .000 | .000 | .001 | .000 | .002 | .000 | .000 | .000 | .000 | .000 | .000 | .000 | .000 | .000 | .000 | .000 | .000 | .006 |  | .000 | .000 | .000 | .000 |
|  | N | 302 | 302 | 302 | 302 | 302 | 302 | 302 | 302 | 302 | 302 | 302 | 302 | 302 | 302 | 302 | 302 | 302 | 302 | 302 | 302 | 302 | 302 | 302 | 302 |
| A21 | Pearson Correlation | .287** | .367** | .329** | .347** | .282** | .336** | .427** | .142* | .412** | .137* | .376** | .361** | .410** | .300** | .403** | .306** | .505** | .393** | .287** | .361** | 1 | .793** | .769** | .578** |
|  | Sig. (2-tailed) | .000 | .000 | .000 | .000 | .000 | .000 | .000 | .013 | .000 | .017 | .000 | .000 | .000 | .000 | .000 | .000 | .000 | .000 | .000 | .000 |  | .000 | .000 | .000 |
|  | N | 302 | 302 | 302 | 302 | 302 | 302 | 302 | 302 | 302 | 302 | 302 | 302 | 302 | 302 | 302 | 302 | 302 | 302 | 302 | 302 | 302 | 302 | 302 | 302 |
| A22 | Pearson Correlation | .248** | .321** | .252** | .296** | .252** | .320** | .389** | .090 | .375** | .073 | .292** | .293** | .350** | .232** | .329** | .253** | .513** | .322** | .205** | .277** | .793** | 1 | .775** | .627** |
|  | Sig. (2-tailed) | .000 | .000 | .000 | .000 | .000 | .000 | .000 | .119 | .000 | .205 | .000 | .000 | .000 | .000 | .000 | .000 | .000 | .000 | .000 | .000 | .000 |  | .000 | .000 |
|  | N | 302 | 302 | 302 | 302 | 302 | 302 | 302 | 302 | 302 | 302 | 302 | 302 | 302 | 302 | 302 | 302 | 302 | 302 | 302 | 302 | 302 | 302 | 302 | 302 |
| A23 | Pearson Correlation | .367** | .396** | .381** | .339** | .313** | .369** | .430** | .229** | .442** | .224** | .370** | .345** | .361** | .283** | .367** | .217** | .485** | .357** | .267** | .300** | .769** | .775** | 1 | .654** |
|  | Sig. (2-tailed) | .000 | .000 | .000 | .000 | .000 | .000 | .000 | .000 | .000 | .000 | .000 | .000 | .000 | .000 | .000 | .000 | .000 | .000 | .000 | .000 | .000 | .000 |  | .000 |
|  | N | 302 | 302 | 302 | 302 | 302 | 302 | 302 | 302 | 302 | 302 | 302 | 302 | 302 | 302 | 302 | 302 | 302 | 302 | 302 | 302 | 302 | 302 | 302 | 302 |
| A24 | Pearson Correlation | .452** | .412** | .427** | .376** | .377** | .354** | .360** | .271** | .420** | .288** | .339** | .328** | .288** | .291** | .301** | .294** | .406** | .285** | .226** | .232** | .578** | .627** | .654** | 1 |
|  | Sig. (2-tailed) | .000 | .000 | .000 | .000 | .000 | .000 | .000 | .000 | .000 | .000 | .000 | .000 | .000 | .000 | .000 | .000 | .000 | .000 | .000 | .000 | .000 | .000 | .000 |  |
|  | N | 302 | 302 | 302 | 302 | 302 | 302 | 302 | 302 | 302 | 302 | 302 | 302 | 302 | 302 | 302 | 302 | 302 | 302 | 302 | 302 | 302 | 302 | 302 | 302 |
| ** Correlation is significant at the 0.01 level (2-tailed).  *Correlation is significant at the 0.05 level (2-tailed). | | | | | | | | | | | |  |  |  |  |  |  |  |  |  |  |  |  |  |  |

Supplementary Table 5. Multicollinearity diagnostics for predictors in the multinomial logistic regression model

Predictor	Tolerance	VIF

| Predictor | Tolerance | VIF |
| --- | --- | --- |
| Sex | 0.745 | 1.343 |
| Age | 0.862 | 1.161 |
| Marital status | 0.859 | 1.164 |
| Place of residence | 0.705 | 1.419 |
| Educational attainment | 0.537 | 1.863 |
| Monthly household income per capita | 0.527 | 1.897 |
| Occupational status | 0.679 | 1.474 |
| Smoking status | 0.823 | 1.215 |
| Alcohol consumption | 0.831 | 1.204 |
| Duration of chronic disease | 0.962 | 1.040 |
